# Asian American Occupational Therapy Practitioners' Perspectives on Supporting the Mental Health of Asian American Caregivers for Older Adults

**DOI:** 10.1155/oti/2063352

**Published:** 2025-07-23

**Authors:** Arianna Bayangos, Rawan AlHeresh, Hadeel R. Bakhsh, Diane Smith

**Affiliations:** ^1^Department of Occupational Therapy, Massachusetts General Hospital Institute of Health Professions, Boston, Massachusetts, USA; ^2^Department of Occupational Therapy, Faculty of Rehabilitation Sciences, University of Jordan, Amman, Jordan; ^3^Department of Community and Preventive Medicine, Public Health Institute, University of Jordan, Amman, Jordan; ^4^Department of Rehabilitation Sciences, College of Health and Rehabilitation Sciences, Princess Nourah Bint Abdulrahman University, Riyadh, Saudi Arabia

**Keywords:** Asian American caregivers, caregiver–provider relationship, mental health, occupational therapy

## Abstract

**Objective:** Asian American (AA) caregivers have unique cultural values that influence their mental health during caregiving. Occupational therapy practitioners (OTPs) are positioned to serve this population because of their holistic lens and their relationship with care recipients and caregivers. The objective of this study was to examine AA OTPs' perspectives on barriers, facilitators, and interventions to support the mental health of AA caregivers in older adults with chronic conditions.

**Design:** This study used a qualitative phenomenological design. Data were collected through virtual interviews (*n* = 10) and focus groups (*n* = 2) and analyzed using thematic analysis.

**Results:** Twelve AA OTPs (*n* = 12) participated in this study, all were occupational therapists (*n* = 12); most were female (*n* = 10) and had a Doctor of Occupational Therapy degree (*n* = 9). Two domains were found during this study: (1) barriers and facilitators to support the mental health of AA caregivers and (2) OTP strategies to support the mental health of AA caregivers. Barriers and facilitators include AA cultural beliefs, provider identity, and access. OTPs can support this population by utilizing their soft skills, assessment and clinical reasoning skills and through interventions, including caregiver education and social support.

**Conclusion:** This study addressed the literature gap on supporting AA caregivers' mental health through an OTP perspective. OTPs can enhance support by understanding AA culture, examining biases, and refining strategies for caregivers' mental health. Healthcare providers can prioritize caregiver support, boost AA representation in healthcare, improve service accessibility, and involve OTPs in caregivers' mental health support.

## 1. Introduction

Over 40 million Americans care for family members aged 50 and older [1]. Caregivers assist with personal needs, household chores, finances, and other tasks. They are crucial to society, providing an average of 22.3 h of care weekly [1]. The increasing elderly population and the complexity of their health needs are making this role more common and demanding [1]. Caregiver burden is the strain caregivers experience [2]. Minority caregivers in the United States, including Asian Americans, face added stressors like language barriers, limited resource knowledge, and discrimination [3, 4]. Asian American caregivers represent 5% of caregivers for older adults [5].

Asian Americans, originating from the “Far East, Southeast Asia, or the Indian subcontinent,” are the fastest-growing ethnic group in the United States, necessitating support for their caregivers due to the high number of older adults requiring care. Despite ethnic diversity, shared cultural values affect their caregiving. Cultural beliefs such as filial piety and utang na loob in Chinese and Filipino cultures emphasize respect and a sense of indebtedness to elders [6, 7], which can heighten caregiver stress [8].

Asian American caregivers often face significant stress, feeling obligated compared to other ethnic groups [9]. The Zarit Burden screening shows that 43% [5] of Asian American caregivers report high strain, higher than White (28.6%), Hispanic/Latino (26.7%), and Black/African American (21. %) [5] caregivers [9]. Contributing factors include higher income and education, linked to cultural obligations to care for family, especially with financial resources [10]. Asian American caregivers tend to be older and have more chronic conditions than noncaregivers [5]. The stress impacts both caregivers' and care recipients' physical and mental health.

In general, for caregivers, high levels of caregiver stress are associated with a higher risk of depression and with higher development and exacerbation of chronic conditions in the caregiver [11]. It is also linked to a higher risk of depression and higher levels of mortality and hospitalization for the care recipient [12, 13]. However, there is limited research conducted specifically on the mental health impacts of caregiving on Asian American caregivers. Asian American caregivers (54%) most frequently interact with healthcare providers compared to other ethnicities; therefore, there is a missed opportunity to support the mental health of this population through healthcare providers, including occupational therapists [14].

The Occupational Therapy Practice Framework: Domain and Process (OTPF-4) outlines how occupational therapy practitioners (OTPs) collaborate with clients and caregivers in decision-making and implementing care plans at home [15]. OTPs' holistic approach considers clients' occupations, environments, life experiences, roles, and physical, cognitive, and social skills, enabling comprehensive support for caregivers' mental health. OTPs assist caregivers with home carryover of therapeutic activities, including daily tasks such as transfers, bathing, grooming, and medication management (OTPF-4). Additionally, OTPs provide caregiver interventions that reduce caregiving time and enhance mental health and coping skills [16].

Spirituality [3, 8, 17–21], social support [22–25], education [25–30], and resilience-building interventions combined with mindfulnes [23, 31–33] were effective in supporting the mental health of Asian caregivers. Psychoeduation [34, 35] and physical activity [36] are potential interventions for this population because of their effectiveness for other caregivers. However, these studies have limitations, such as most studies focusing on East Asia, limited generalizability to Southeast Asia and the Indian subcontinent, the limited number of intervention studies performed on the Asian American caregiver population, and the heavy emphasis on caregivers to those with dementia in the literature.

Research must address the gap in supporting Asian American caregivers' mental health due to the rising number of elderly Asian Americans, their unique cultural values, and the high stress levels they endure. While existing research has explored the experiences of Asian American caregivers [3, 37–39], few studies have examined the insights of providers who work closely with this population. In particular, OTPs, who share the same cultural background, may offer a distinct and valuable lens through which to understand the nuanced mental health needs of Asian American caregivers. Racial and cultural concordance between healthcare providers and patients can improve trust, communication, and patient satisfaction [40]. Asian American OTPs may be more attuned to the cultural expectations, language nuances, and intergenerational dynamics that shape caregiving in Asian families—insights that non-Asian American providers may not fully access or interpret.

Focusing on Asian American OTPs allows this study to explore how shared cultural identity and lived experiences influence therapeutic relationships, provider empathy, and culturally attuned intervention strategies. This perspective may reveal barriers and facilitators that are invisible in caregiver-centered research. Therefore, this study is aimed at addressing this gap by interviewing Asian American OTPs regarding barriers, facilitators, and interventions aiding the mental health of these caregivers. By interviewing practitioners rather than caregivers, the study offered insight into recurring patterns, cultural dynamics, and systemic barriers that caregivers may hesitate to disclose directly due to mental health stigma in many Asian communities [8]. Additionally, cultural concordance between OTPs and the population of interest enhances interpretive validity, as shared cultural understanding enables deeper recognition of caregiving norms, communication styles, and emotional burdens [41].

## 2. Methods

### 2.1. Study Design

This study used a phenomenological qualitative design to assess what Asian American OTPs view as barriers, facilitators, and effective interventions to support Asian American family caregivers via focus groups and individual interviews [42].

### 2.2. Participants, Recruitment, and Sampling

Participants were recruited via convenience and snowball sampling, including contacting OTPs via social media, word of mouth, posting the study on the American Occupational Therapy Association's (AOTA) CommunOT, an online platform for AOTA members, and sharing the study on social media platforms, including the Coalition of Occupational Therapy Advocates for Diversity (COTAD) and Association of Asian Americans and Pacific Islanders in Occupational Therapy (AAPI-OT), from February to March 2024.

Eligible participants were required to (1) identify as Asian American, (2) be licensed OTPs, (3) have experience working with Asian American older adults with chronic conditions and their families, (4) possess at least 1 year of experience in adult physical dysfunction settings such as skilled nursing facilities, inpatient rehabilitation, acute care, home health, and outpatient settings, (5) be able to commit 1–2 h for the study (including focus group/individual interview and an optional member check), and (6) have reliable Internet access and a device for video conferencing. Those not meeting all criteria were excluded. Interested participants completed a Microsoft Form or contacted the research team via email or social media.

### 2.3. Data Collection

Qualitative data were collected through a 60-min virtual focus group and 30-min virtual individual interviews from February to March 2024. Focus groups and individual interviews were the chosen method of data collection to gather more in-depth information regarding OTPs' experiences with Asian American caregivers and to give participants opportunities to hear the views of others. The focus groups and individual interviews were conducted concurrently based on the availability of the participants. Participants chose between a focus group or an individual interview, resulting in 10 interviews (*n* = 10) and one focus group of 2 participants (*n* = 2). The focus group originally included three participants, but one participant changed to an individual interview due to their availability. The two participants in the focus group wanted to continue to participate in a focus group rather than an individual interview. One individual interview participant provided a supporting written file for additional elaboration. The interview guide (Appendix 1), developed from current literature, included questions to explore Asian American OTPs' experiences with Asian American caregivers, as well as barriers, facilitators, and interventions for caregivers' mental health. Interviews were audiorecorded and transcribed via secured Zoom through Massachusetts General Brigham.

### 2.4. Data Analysis

Demographic data were analyzed to determine OTPs' ages, genders, education levels, work settings, states of practice, Asian American subethnicities, years of experience, and the percentage of Asian Americans on their caseload. Qualitative data were analyzed through Braun et al.'s reflexive thematic analysis to identify themes related to barriers, facilitators, and effective interventions for supporting Asian American caregivers [43].

The research assistant read all transcripts twice before creating an inductively developed codebook with operational definitions for each theme and subtheme. Interview and focus group data were coded line-by-line using Dedoose, with a second team member reviewing the initial codes for confirmability. Participants in the focus group and individual interviews were optionally involved in a member check, where they received a document outlining the domains, themes, and subthemes and had 1 week to provide feedback. Six participants completed the member check, all agreeing with the provided themes.

### 2.5. Ethics

Ethical approval was granted on February 5, 2024, by the Mass General Brigham Institutional Review Board (Protocol #: 2023P002648). Occupational therapists were informed of the study's aims and potential risks. Participation was voluntary, and verbal informed consent was obtained prior to individual interviews and the focus group.

### 2.6. Research Team and Reflexivity

The first author (A.B.), a female occupational therapist student, conducted recruitment, data collection, and analysis for this study and has been researching the mental health support of Asian American caregivers for older adults with chronic conditions since September 2022. The research team included three female academic occupational therapists, R.A., H.R.B., D.S. experienced in methodology, outcome measures, and research on health conditions. The first author, a Filipino American, facilitated participants' comfort in sharing sensitive cultural experiences. There was no prior relationship between the participants and the first author, though her educational background and personal experience may have influenced the project.

## 3. Results

Twelve Asian American OTPs (*n* = 12) participated in this study who were mostly female (*n* = 10), had a doctorate of occupational therapy (OTD) degree (*n* = 9), and had 0%–25% of Asian Americans on their caseload (*n* = 8). A detailed description of the participant demographics is provided in Table 1. There were 10 individual interviews and one focus group with two participants..

Two domains were developed by analyzing interview and focus group responses: (1) barriers and facilitators to support the mental health of Asian American caregivers and (2) OTP strategies to support the mental health of Asian American caregivers.

### 3.1. Domain 1: Barriers and Facilitators Supporting the Mental Health of Asian American Caregivers

Within barriers and facilitators to support the mental health of Asian caregivers, themes included (1) Asian American cultural beliefs, (2) provider identity, and (3) access (Table 2).

#### 3.1.1. Asian American Cultural Beliefs

Many participants described the importance of incorporating Asian American cultural beliefs in supporting the mental health of Asian American caregivers. Four subthemes were described under Asian American cultural beliefs, including (1) different perspectives within the Asian American umbrella, (2) family and caregiving, (3) image, and (4) beliefs about mental health.

One participant emphasized the importance of disaggregating Asian American subgroups to better address caregivers' needs, noting varying views on filial obligations, spirituality, health, wellness, and death. Moreover, participants argued against a one-size-fits-all approach for all subgroups. Additionally, participants highlighted similarities within each Asian culture about views on families and caregiving, but equally highlighted differences within each Asian culture in how families adapt to American culture.

Participants discussed how Asian American families are typically multigenerational, and there is an expectation that families will take care of one another as they age or become ill: “Even now when I go to India, […] it's a very collective community mindset. […] Everybody kind of rallies together and takes care of each other.” A participant also shared that with this community mindset, there can be unidentified caregivers as there may be many family members involved in the care recipients' care. Some participants stated that within some Asian cultures, there is a reverence toward the elderly, and this dynamic can affect how families view caregiving. The older adult care recipient has a significant say in the caregiving process, with some participants sharing that for Asian older adults, there is a preference to be taken care of by family, and that they may be uncomfortable with outside help.

Participants shared that younger Asian caregivers in the sandwich generation, who care for both elderly family members and children [42], experience greater difficulty due to multiple responsibilities. They have also expressed a cultural and generational mismatch, indicating a preference for providing financial or emotional support rather than physical care.

Participants also highlighted the significance of one's public image. Some noted that acknowledging mental health struggles might be seen as a sign of weakness. One participant mentioned, “My own mother [...] would embellish stories to appear favorable to her friends. [...] She doesn't want to be perceived as odd or weak.”

Participants discussed their beliefs about mental health in the Asian American community, noting that it is often not addressed. However, many observed a growing awareness of mental health issues across both younger and older generations. One participants discussed “We are making strides, and people are willing to share about mental health. I almost feel like everyone is […] struggling with something.” “[My parents are] in their early seventies now, so I've had some close family members deal with very severe mental health problems, and that made my parents [realize] that those things can't really be controlled.”

#### 3.1.2. Provider Identity

Participants talked about how the identity of a provider can be a facilitator or barrier to supporting the mental health of Asian American caregivers with (1) Asian American OTPs and caregivers' shared identity and (2) bias of a provider.

Some participants talked about the mutual connections between them and their caregivers. “[Caregivers] feel more seen and heard from somebody who is also Asian American.” Some participants also discussed how providers' biases, including the minority myth (the perception that Asian Americans are hardworking, have achieved upward social mobility, and are free from hardships like racism and discrimination) [44], could negatively affect working with caregivers. One participant shared that providers may falsely believe that caregivers who do not speak English are not smart and, therefore, cannot have deeper conversations with these caregivers about topics such as mental health.

#### 3.1.3. Access

Outside of the caregiver and the provider, participants discussed how access could be a barrier or facilitator to support the mental health of Asian American caregivers, which includes (1) the environment, (2) language, and (3) accessing healthcare.

Some participants discussed how the environment could lead to either a lack or an abundance of caregiver resources. One participant discussed how he lived in a neighborhood with many Asian Americans; therefore, there were more resources for this group: “But what if […] somebody who's like living in a white neighborhood? [What if] is the family like speaking an Asian language? And then there aren't that many resources around them.”

Participants noted that language significantly impacts caregivers' mental health. They mentioned that healthcare providers speaking the same language as caregivers increased comfort. However, they faced challenges in communication and resource provision due to limited resources in certain Asian languages. Additionally, some multilingual caregivers had the added responsibility of translating.

Participants highlighted the United States healthcare system's emphasis on provider productivity, neglecting caregivers' mental health: “It's easier to focus solely on the patient and overlook the systems, including caregivers.” They also noted underutilization of psychology services for caregivers: “There was no guidance for mental health when I made referrals and concerns. There was no response.”

Participants highlighted the crucial role of resources such as support groups and respite care in aiding caregivers' mental health. However, they noted the difficulty in locating and affording these resources due to financial limitations. One participant mentioned, “When you're caregiving and facing high demands, you lack the energy to search for resources.”

### 3.2. Domain 2: OTP Strategies to Support the Mental Health of Asian American Caregivers

The participants described how OTPs' actions and OTP interventions could facilitate the mental health of Asian American caregivers (Table 3).

#### 3.2.1. OTP's Actions

Participants discussed how utilizing their (1) soft skills and (2) assessment skills and clinical reasoning could facilitate mental health for caregivers.

Many participants talked about building rapport with and prioritizing caregivers: ‘If a patient is occupied with an activity, and I know that they are safely doing it, […] I've had caregivers ask to talk to me on the side and they'll chat with me about their fears.' Other participants talked about the importance of validating what caregivers were going through, including sharing that they had seen these difficulties in other families and giving them permission to take care of their mental health.

Participants discussed how they used their assessment skills and clinical reasoning to gauge whether the caregiver was equipped to care for their care recipient and whether there were differences in the caregivers and care recipients' expectations for care: “Do you have the capacity to provide the level of care that this person needs? […] Capacity means a few different things […] physical capacity, emotional capacity, and the mental capacity, like the bandwidth to actually help.” Participants also discussed the importance of gauging caregivers' comfort while discussing mental health, their risk of decreased mental health, and their comfort when discussing support resources. In addition, a participant discussed how OTPs can help families with care planning to determine what caregiving will look like for families before their family members age and require more care.

#### 3.2.2. OTP Interventions

Many participants discussed the importance of family involvement in the therapy process by asking them to help complete the occupational profile of their care recipient, showing them their care recipient's progress, and helping them become comfortable with the discharge home.

“Family involvement […] can […] help garner trust between the family and therapists, or like people making recommendations that might address their mental health.”

One participant stated that she integrated valued occupations with the caregiver, including making dumpling, to boost the care recipient's physical outcomes and the secondary outcome of increasing mental health in the care recipient and caregiver.

Many participants discussed how they educate caregivers on the care recipient's condition and the recovery process, how to perform skills such as transfers safely, and how caregivers can take care of themselves. One participant discussed how she personalized the handouts for each caregiver when she provided caregiver education.

The participants discussed how they either led caregiver support groups or referred caregivers to other groups. Participants talked about how support groups could be a place for caregivers to freely express their feelings.

“Because there was a group […] specifically designated and focused for caregivers to […] express […] their frustrations […] they don't want to feel like they're […] encroaching […] their relatives'[…] time and treatment and space.”

Participants talked about the importance of utilizing interprofessional collaboration and including the caregiver as a member of the team. Participants stated that they collaborated with other healthcare professionals, including social workers, nurses, speech-language pathologists, aging specialists, and doctors to communicate caregivers' mental health concerns and determine how to support them.

Some participants discussed how they referred caregivers to community organizations that caregivers may trust, including consulates and places of worship. A participant stated, “With the help of […] an organization being able to promote more messages along, mental health can be very empowering, and really […] destigmatized.” One participant shared that adult day health programs, where there are different healthcare providers in one place to support the care recipient, can also be beneficial for caregivers.

Some participants discussed how addressing mental health is an emerging area in OTPs. A participant stated that occupational therapy is not utilized enough in mental health, “A lot of people don't know that OT practitioners have a mental health training, or they just assume if they want mental health, then they'll […] get a psych consult.”

## 4. Discussion

This study examined Asian American OTPs' views on barriers, facilitators, and interventions to support Asian American caregivers' mental health. Results indicate that understanding Asian American culture, including beliefs, languages, and shared identities between providers and caregivers, is crucial. The study offers the unique perspective of Asian American OTPs, skilled in analyzing how context and culture affect caregiving and sharing an identity with this population, unlike existing literature which focuses on the caregiver's perspective.

A participant emphasized the importance of avoiding a “fix-all methodology” for every Asian American ethnic subgroup (e.g., Filipino, Chinese, and Taiwanese) due to cultural and assimilation differences. Thus, OTPs and healthcare providers should inquire about caregivers' specific cultural beliefs and their influence on caregiving practices. Nevertheless, participants noted common themes in Asian American culture affecting caregiving.

Similar to other studies, [10, 45] this study found that Asian American caregivers are very involved in caregiving and that there are negative and positive consequences [23, 46] of this high level of family involvement. However, this study adds to current knowledge by addressing how OTPs can navigate this cultural value. This can include care planning (helping families determine the younger and older generations' expectations for caregiving) and gauging what level of support families can provide, including physical support (activities of daily living, including transfers and toileting), emotional support, and other support (financial support and finding resources for the main caregiver). OTPs can then provide resources to supplement the help caregivers provide to their loved ones and help caregivers manage their daily routine. With multiple family members involved in the caregiving process, one participant shared that this could lead to unidentified caregivers whom healthcare providers may not target for mental health support. Meyer et al. discussed how Vietnamese American families and healthcare professionals working with this community believed that it is important to integrate all of the family members involved in caregiving duties to improve caregiver mental health [19]. For this population, it is essential for OTPs to ask who is involved in caregiving duties to provide mental health support to all involved.

A study found that negative attitudes toward mental health could increase caregiver burden [46]. Therefore, this study offers practical recommendations for OTPs and healthcare providers, emphasizing soft skills and assessment abilities. These include building client trust and identifying cues of caregiver mental health risks, such as rent and medical expense difficulties. Participants suggested indirectly asking mental health-related questions using terms like “worried,” “scared,” “lack of enjoyment in life,” and “fatigue.” They also stressed the importance of culturally sensitive mental health education, focusing on Asian American values and family priorities.

Moreover, a study found that non-English-speaking Asian caregivers experience more distress than their English-speaking counterparts due to limited non-English resources and the additional burden of translating for their loved ones [47]. This study was able to dive into the possible reasoning behind this, including insufficient resources in languages other than English and the added responsibility of multilingual caregivers to act as translators for their loved ones. The healthcare system must provide adequate multilingual resources, including interpreters, to address language barriers that lead to miscommunication and poorer health outcomes [48]. Providers should utilize these resources instead of relying on caregivers for translation, allowing caregivers to engage fully in care planning and express mental health concerns.

The literature lacks insight into how the relationship between an OTP and an Asian American caregiver influences caregiver mental health. This study highlights the necessity of Asian American OTPs in the workforce to better serve the increasing number of Asian American older adults and their caregivers. These OTPs offer a shared cultural understanding in therapeutic relationships. This study revealed biases among OTPs, such as the assumption that all Asian American families will care for their loved ones and the model minority myth (the perception that Asian Americans are hardworking, have achieved upward social mobility, and are free from hardships like racism and discrimination) [49]. These biases could lead healthcare providers to overlook Asian American caregivers, assuming they are not prone to mental health issues. Consequently, it is crucial for OTPs to understand Asian American culture and to ask caregivers open-ended questions about their experiences and beliefs.

In addition to building rapport and using their assessment skills, the participants also discussed caregiver intervention strategies. Like other studies, caregiver education [25–30] and social support [22–25] were popular intervention strategies used by participants. This study provides suggestions on how to support this population. One of the common findings of this study was how OTPs can have a greater role in supporting the mental health of Asian American caregivers because of how OTPs spend more time with caregivers than with other healthcare providers and, therefore, can create more intimate relationships with caregivers and care recipients according to the participants of this study. Therefore, OTPs can advocate for their role as mental health providers by explaining the training provided in the occupational therapy curriculum and how OTPs consider how personal and environmental factors can impact the role of caregiving and caregiver mental health [15]. Overall, this study provides perspectives on the important cultural values that OTPs should consider when working with this population, systemic barriers that may hinder supporting the mental health of this population, and OTP strategies for supporting the mental health of this population.

### 4.1. Strengths and Limitations

This study presented new literature on the underrepresented yet increasing group of Asian American caregivers through the perspective of Asian American OTPs, emphasizing significant cultural values and the influence of provider identity on these caregivers. Participants included individuals from the Far East (Chinese and Taiwanese), Southeast Asia (Filipino, Thai, and Vietnamese), and the Indian subcontinent (Indian) [6].

This study, despite offering new insights into the literature on this population, has limitations. It included only occupational therapists, excluding occupational therapy assistants, whose perspectives should be considered in future research. The sample had more Southeast Asian Americans (*n* = 8) than participants from the Far East (*n* = 3) and the Indian subcontinent (*n* = 1). Future studies should include a more diverse sample, particularly from the Indian subcontinent. Conducted over a semester from January to April 2024, the study's small sample size (*n* = 12) still reached data saturation, with no new codes emerging in the final interview. The author conducted and coded all interviews, creating themes, and has 2 years of related research experience and shares an identity with the population, aiding data interpretation. Another team member reviewed the themes, and participants verified them through an optional member check.

This study suggests future research should include randomized controlled trials of occupational therapy interventions used by participants, such as education, social support, and referrals to trusted community organizations. Additionally, qualitative studies on Asian American caregivers' experiences with OTPs supporting their mental health would be beneficial.

## 5. Conclusion

This study investigated Asian American OTPs' views on barriers, facilitators, and interventions for supporting the mental health of Asian American caregivers. Barriers and facilitators include Asian cultural values, provider identity, and access. OTPs' strategies for aiding caregivers' mental health entail soft skills, assessment skills, clinical reasoning, integrating caregivers into patient sessions, caregiver education, support groups, interprofessional collaboration, and referrals to trusted community organizations. Participants noted that addressing caregivers' mental health is an emerging practice area.

Therefore, OTPs should understand Asian American cultural values, reflect on their biases, assess their skills and interventions, and advocate for OTPs' role in supporting caregivers' mental health. Systemic changes are needed in healthcare, such as recognizing the importance of caregiver support, increasing Asian American representation in healthcare professions, offering accessible services nationwide in various languages, and integrating OTPs in caregiver mental health support.

## Figures and Tables

**Table 1:** Demographic data of occupational therapy practitioners (*n* = 12).

	**N**	**(%)**	**M(SD)**
Age (*n* = 12)			34.82 (13.10)
18–24	2	16.67%	
25–34	6	50%	
35–44	2	16.67%	
45+	1	8.33%	
Prefer not to say	1	8.33%	
Gender (*n* = 12)			
Female	10	83.33%	
Male	2	16.67%	
Education (*n* = 12)			
Bachelor's degree	2	16.67%	
Master's degree	1	8.33%	
OTD	9	75%	
Settings (*n* = 12)			
Academia	2	5.1%	
Acute care (adult)	5	12.8%	
Assisted living	2	5.1%	
Consulting	1	2.6%	
Home health	5	12.8%	
Inpatient mental health	1	2.6%	
Inpatient rehabilitation	6	15.38%	
Outpatient adult	7	17.9%	
Pediatrics	3	7.7%	
Postacute care	1	2.6%	
Skilled nursing facility	5	12.8%	
Vocational rehabilitation	1	2.6%	
States (*n* = 12)			
CA	8	66.67%	
IL	1	8.33%	
MA	1	8.33%	
MD	1	8.33%	
NJ	1	8.33%	
Race within Asian American umbrella (*n* = 12)			
Chinese	2	16.67%	
Filipino	5	41.67%	
Indian	1	8.33%	
Taiwanese	1	8.33%	
Thai	1	8.33%	
Vietnamese	2	16.67%	
Years of experience as an occupational therapy practitioner (*n* = 12)			9.54 (13.85)
1–4	6	50%	
5–9	2	16.67%	
10–14	2	16.67%	
14–19	1	8.33%	
20+	1	8.33%	
Percentage of caseload that is Asian American (*n* = 12)			
0%–25%	8	66.67%	
26%–50%	3	25%	
51%–75%	1	8.33%	
75%–100%	0	0%	

**Table 2:** Domain 1: Barriers and facilitators supporting the mental health of Asian American caregivers.

**Domain**	**Theme**	**Subthemes**
Barriers and facilitators to supporting the mental health of Asian American caregivers	Asian American cultural beliefs	Different perspectives within the Asian American umbrella
		Family and caregiving
		Image
		Beliefs about mental health
	Provider identity	Asian American OTPs and Asian American caregivers' shared identity
		Bias
	Access	Environment
		Language
		Accessing healthcare

**Table 3:** Domain 2: OTP strategies to support the mental health of Asian American caregivers.

**Domain**	**Theme**	**Subthemes**
OTP strategies to support the mental health of Asian American caregivers	OTP's actions	Soft skills
		Assessment skills and clinical reasoning
	OTP interventions	Integration of caregiver into patient sessions
		Caregiver education
		Support groups
		Interprofessional collaboration
		Referral to trusted community organizations
		Addressing mental health is an merging area

## Data Availability

The data that support the findings of this study are available on request from the corresponding author. The data are not publicly available due to privacy or ethical restrictions.

## Appendix 1

Focus group and individual interview questions:

Demographic data collected:

• Age
• State
• Education level
• Gender
• What ethnicity do you identify as within the Asian American umbrella?
• What settings do you currently work in and have worked in in the past?
• How many years of experience do you have as an occupational therapy practitioner?
• How many patients do you see a week? What percentage of them are Asian American?

Age, state, and education level were optional for the participants.

Focus group and individual interview questions:

• A brief description of the experiences of working with Asian American older adults and their family caregivers.
• What does mental health mean to the Asian American population?
• What are the facilitators to successfully support the mental health of Asian American caregivers?
• What are the barriers to successfully supporting the mental health of Asian American caregivers?
• Describe the interventions used to support Asian American family caregivers and their mental health.
• Is there anything else that you would like to add about this topic that we have not discussed?

## References

[1] American Association of Retired Persons (AARP) (2020). *Caregiving in the United States 2020*.

[2] Liu Z., Heffernan C., Tan J. (2020). Caregiver Burden: A Concept Analysis. *International Journal of Nursing Sciences*.

[3] Tran J. T., Theng B., Tzeng H. M. (2023). Cultural Diversity Impacts Caregiving Experiences: A Comprehensive Exploration of Differences in Caregiver Burdens, Needs, and Outcomes. *Cureus*.

[4] Loo Y. X., Yan S., Low L. L. (2022). Caregiver Burden and Its Prevalence, Measurement Scales, Predictive Factors and Impact: A Review With an Asian Perspective. *Singapore Medical Journal*.

[5] Miyawaki C. E., Bouldin E. D., Taylor C. A., McGuire L. C., Markides K. S. (2023). Characteristics of Asian American Family Caregivers of Older Adults Compared to Caregivers of Other Racial/Ethnic Groups: Behavioral Risk Factor Surveillance System 2015–2020. *Journal of Applied Gerontology*.

[6] Wakui T., Cheng S.-T. (2023). Well-Being and Filial Piety. *Encyclopedia of Quality of Life and Well-Being Research*.

[7] Pan Y., Chen R., Yang D. (2022). The Relationship Between Filial Piety and Caregiver Burden Among Adult Children: A Systematic Review and Meta-Analysis. *Geriatric Nursing*.

[8] Huang Y.-h., Nagao C. A., Santos K. M. B., Werchowsky M. I. (2022). Impact of Culture, Spirituality, and Mental Health Attitudes on Intergenerational Asian-American Caregivers: A Pilot Study. *American Journal of Occupational Therapy*.

[9] Whitney R. L., Bell J. F., Kilaberia T. R. (2023). Diverse Demands and Resources Among Racially/Ethnically Diverse Caregivers. *Ethnicity & Health*.

[10] Meyer O. L., Liu X., Nguyen T.-N., Hinton L., Tancredi D. (2018). Psychological Distress of Ethnically Diverse Adult Caregivers in the California Health Interview Survey. *Journal of Immigrant and Minority Health*.

[11] Broxson J., Feliciano L. (2020). Understanding the Impacts of Caregiver Stress. *Professional Case Management*.

[12] Ejem D., Bauldry S., Bakitas M., Drentea P. (2018). Caregiver Burden, Care Recipient Depressive Symptomology, and Social Exchange: Does Race Matter?. *Journal of Palliative Care*.

[13] Kuzuya M., Enoki H., Hasegawa J. (2011). Impact of Caregiver Burden on Adverse Health Outcomes in Community-Dwelling Dependent Older Care Recipients. *The American Journal of Geriatric Psychiatry*.

[14] Montenegro X. (2014). *Caregiving among Asian Americans and Pacific Islanders Age 50+*.

[15] Boop C., Cahill S. M., Davis C. (2020). Occupational Therapy Practice Framework: Domain and Process Fourth Edition. *AJOT: American Journal of Occupational Therapy*.

[16] Sörensen S., Pinquart M., Duberstein P. (2002). How Effective Are Interventions With Caregivers? An Updated Meta-Analysis. *An Updated Meta-Analysis. The Gerontologist*.

[17] Badana A. N. S., Andel R. (2018). Aging in the Philippines. *Gerontologist*.

[18] Alviar S., del Prado A. (2022). “You Should Pray About It”: Exploring Mental Health and Help-Seeking in Filipino American Catholics. *Asian American Journal of Psychology*.

[19] Meyer O. L., Fukurai M., Ho J. (2020). Dementia Caregiver Intervention Development and Adaptation in the Vietnamese American Community: A Qualitative Study. *Dementia*.

[20] Tyagi S., Luo N., Tan C. S. (2023). Qualitative Study Exploring Heterogeneity in Caregiving Experiences Post-Stroke in Singapore. *BMJ Open*.

[21] Rahayu H., Yona S., Masfuri (2021). The Social Support, Spirituality, Stress, and Family Burden of Cancer Patients in Jakarta Hospitals. *Enfermería Clínica*.

[22] Biclar J. E. D. P., Tan-Mansukhani R., Simon P. D. (2022). Self-Efficacy and Psychological Well-Being of Family Caregivers of Persons With Spinal Cord Injury. *Psychological Studies*.

[23] Zhong X., Song P. P., Wang Z., Chen H. (2022). Resilience Building Among Chinese Family Caregivers of Older People With Parkinson’s Disease in Shanghai. *Health & Social Care in the Community*.

[24] Han E.-J., Park M., Park S., Giap T. T. T., Han D. (2020). Randomized Controlled Trial of the Caregiver Orientation for Mobilizing Personal Assets and Strengths for Self-Care (COMPASS) for Caregiving Journey: A National Family Caregiver Support Program in a Long-Term Care Insurance System. *Journal of the American Medical Directors Association*.

[25] Chen H.-M., Huang M.-F., Yeh Y.-C., Huang W. H., Chen C. S. (2015). Effectiveness of Coping Strategies Intervention on Caregiver Burden Among Caregivers of Elderly Patients With Dementia. *Psychogeriatrics*.

[26] Yang S.-Y., Fu S.-H., Hsieh P.-L., Lin Y. L., Chen M. C., Lin P. H. (2022). Improving the Care Stress, Life Quality, and Family Functions for Family-Caregiver in Long-Term Care by Home Modification. *Industrial Health*.

[27] Chen W., Ma H., Wang X., Chen J. (2020). Effects of a Death Education Intervention for Older People With Chronic Disease and Family Caregivers: A Quasi-Experimental Study. *Asian Nursing Research*.

[28] Hinton L., Tran D., Nguyen T.-N., Ho J., Gitlin L. (2019). Interventions to Support Family Caregivers of People Living With Dementia in High, Middle and Low-Income Countries in Asia: A Scoping Review. *BMJ Global Health*.

[29] Baruah U., Varghese M., Loganathan S. (2021). Feasibility and Preliminary Effectiveness of an Online Training and Support Program for Caregivers of People With Dementia in India: A Randomized Controlled Trial. *International Journal of Geriatric Psychiatry*.

[30] Lee J.-A., Nguyen H., Park J., Tran L., Nguyen T., Huynh Y. (2017). Usages of Computers and Smartphones to Develop Dementia Care Education Program for Asian American Family Caregivers. *Hir*.

[31] Palacio G. C., Krikorian A., Gómez-Romero M. J., Limonero J. T. (2020). Resilience in Caregivers: A Systematic Review. *American Journal of Hospice and Palliative Medicine*.

[32] Ahn S.-H., Kim S. H. (2022). Distress, Family Resilience, and Quality of Life Among Family Caregivers of Cancer Patients Undergoing Chemotherapy: The Moderating Role of Family Resilience. *Korean Journal of Adult Nursing*.

[33] Lun M. W. A. (2019). The Effectiveness of a Life Story Program on Stress Reduction Among Chinese American Family Caregivers of Older Adults. *Educational Gerontology*.

[34] Cheng S.-T., Li K.-K., Losada A. (2020). The Effectiveness of Nonpharmacological Interventions for Informal Dementia Caregivers: An Updated Systematic Review and Meta-Analysis. *Psychology and Aging*.

[35] Rouch S. A., Fields B. E., Alibrahim H. A., Rodakowski J., Leland N. E. (2021). Evidence for the Effectiveness of Interventions for Caregivers of People With Chronic Conditions: A Systematic Review. *American Journal of Occupational Therapy*.

[36] Marshall E., LaCaille R. A., LaCaille L. J., Lee J. E., Peterson E. (2022). Effects of Physical Activity Interventions for Caregivers of Adults: A Meta-Analysis. *Health Psychology*.

[37] Han M., Diwan S., Sun K. (2019). Exploring Caregiving-Related Experiences Among Chinese American and European American Family Caregivers of Persons With Mental Illness. *Transcultural Psychiatry*.

[38] Raj M., Zhou S., Yi S. S., Kwon S. (2021). Caregiving Across Cultures: Priority Areas for Research, Policy, and Practice to Support Family Caregivers of Older Asian Immigrants. *American Journal of Public Health*.

[39] Choi J., Van T. P., Edward N., Jung A., Tsoh J. (2024). Understanding the Caregiving Experiences of Asian Indian, Chinese, Korean, and Vietnamese American Family Care Partners of Persons Living With Dementia. *Aging & Mental Health*.

[40] Shen M. J., Peterson E. B., Costas-Muñiz R. (2018). The Effects of Race and Racial Concordance on Patient-Physician Communication: A Systematic Review of the Literature. *Journal of Racial and Ethnic Health Disparities*.

[41] Kagawa-Singer M., Dressler W., George S., Elwood W. N. (2015). *The Cultural Framework for Health*.

[42] Portney L. G. (2020). *Foundations of Clinical Research: Applications to Evidence-Based Practice*.

[43] Braun V., Clarke V., Hayfield N., Terry G. (2019). Thematic Analysis. *Handbook of Research Methods in Health Social Science*.

[44] Mc Kittrick A., Gustafsson L., Marshall K. (2021). A Systematic Review to Investigate Outcome Tools Currently in Use for Those With Hand Burns, and Mapping Psychometric Properties of Outcome Measures. *Burns*.

[45] Isac C., Lee P., Arulappan J. (2021). Older Adults With Chronic Illness – Caregiver Burden in the Asian Context: A Systematic Review. *Patient Education and Counseling*.

[46] Huang Y.-h., Nagao C., Michelle Santos K., Werchowsky M. (2021). Asian-American Caregivers: Factors That Influence Caregiver Burden. *American Journal of Occupational Therapy*.

[47] Nguyen J. P., Hoang D., Zhou K., Harvey D. J., Meyer O. L. (2020). Impact of Acculturation on Psychological Distress in Latino and Asian Caregivers. *Alzheimer's & Dementia*.

[48] Al Shamsi H., Almutairi A. G., Al Mashrafi S., Al Kalbani T. (2020). Implications of Language Barriers for Healthcare: A Systematic Review. *Oman Medical Journal*.

[49] Le T. P., Lee E. A., Bansal P., Shin R. Q. (2024). Internalized Model Minority Myth, Distress, and Anti-Black Attitudes Among Low-Income Asian Americans. *Asian American Journal of Psychology*.

